# The biting rate of *Aedes aegypti* and its variability: A systematic review (1970–2022)

**DOI:** 10.1371/journal.pntd.0010831

**Published:** 2023-08-08

**Authors:** Mondal Hasan Zahid, Hannah Van Wyk, Amy C. Morrison, Josefina Coloma, Gwenyth O. Lee, Varsovia Cevallos, Patricio Ponce, Joseph N. S. Eisenberg

**Affiliations:** 1 Department of Epidemiology, School of Public Health, University of Michigan, Ann Arbor, Michigan, United States of America; 2 Department of Biology, University of Florida, Gainesville, Florida, United States of America and Emerging Pathogens Institute, University of Florida, Gainesville, Florida, United States of America; 3 Department of Pathology, Microbiology, and Immunology, University of California Davis, Davis, California, United States of America; 4 Division of Infectious Diseases and Vaccinology, School of Public Health, University of California Berkeley, Berkeley, California, United States of America; 5 Rutgers Global Health Institute & Department of Biostatistics and Epidemiology, School of Public Health, Rutgers, The State University of New Jersey, Newark, New Jersey, United States of America; 6 Instituto Nacional de Investigación en Salud Pública, Centro de investigación en enfermedades infecciosas y vectoriales-CIREV, Quito, Ecuador; Radboud University Nijmegen Radboud Institute for Molecular Life Sciences: Radboud Universiteit Radboud Institute for Molecular Life Sciences, NETHERLANDS

## Abstract

**Background:**

Transmission models have a long history in the study of mosquito-borne disease dynamics. The mosquito biting rate (MBR) is an important parameter in these models, however, estimating its value empirically is complex. Modeling studies obtain biting rate values from various types of studies, each of them having its strengths and limitations. Thus, understanding these study designs and the factors that contribute to MBR estimates and their variability is an important step towards standardizing these estimates. We do this for an important arbovirus vector *Aedes aegypti*.

**Methodology/Principal findings:**

We perform a systematic review using search terms such as ‘biting rate’ and ‘biting frequency’ combined with ‘*Aedes aegypti’* (‘*Ae*. *aegypti’* or ‘*A*. *aegypti’*). We screened 3,201 articles from PubMed and ProQuest databases, of which 21 met our inclusion criteria. Two broader types of studies are identified: human landing catch (HLC) studies and multiple feeding studies. We analyze the biting rate data provided as well as the methodologies used in these studies to characterize the variability of these estimates across temporal, spatial, and environmental factors and to identify the strengths and limitations of existing methodologies. Based on these analyses, we present two approaches to estimate population mean per mosquito biting rate: one that combines studies estimating the number of bites taken per gonotrophic cycle and the gonotrophic cycle duration, and a second that uses data from histological studies. Based on one histological study dataset, we estimate biting rates of *Ae*. *aegypti* (0.41 and 0.35 bite/mosquito-day in Thailand and Puerto Rico, respectively).

**Conclusions/Significance:**

Our review reinforces the importance of engaging with vector biology when using mosquito biting rate data in transmission modeling studies. For *Ae*. *aegypti*, this includes understanding the variation of the gonotrophic cycle duration and the number of bites per gonotrophic cycle, as well as recognizing the potential for spatial and temporal variability. To address these variabilities, we advocate for site-specific data and the development of a standardized approach to estimate the biting rate.

## Author summary

Half of the world’s population is now at risk of dengue infection, which transmits to humans mostly through the bite of an infected female *Aedes aegypti* mosquito. Disease transmission models have played an important role in understanding the dynamics of dengue transmission and helping to develop control measures. The mosquito biting rate is one of the central parameters used in these models. Mosquito biting rates used in existing works are taken from a variety of studies, each with its strengths and limitations. To understand how existing study designs are used to estimate biting rate and how these estimates may vary over time, space, and environmental factors, we perform a systematic review of biting rate studies. We identify three study designs (human landing catch, marked-release-recapture, and histological) that play an important role in estimating per mosquito biting rates and capturing variability across a number of environmental factors. In particular, human landing catch studies can capture the variability of biting rates and marked-released-recapture studies along with histological studies can quantify the multiple feeding that occurs between ovipositions. Transmission modeling studies should be more informed by the biology of mosquito behavior. By understanding the biology of blood-feeding and context-specific factors, we can arrive at more informed per mosquito biting rate estimates for site-specific transmission model analysis.

## Introduction

Mosquitoes are vectors for an array of infectious diseases such as malaria, dengue, West Nile fever, yellow fever, Zika, chikungunya, and lymphatic filariasis [1]. The pathogens causing these diseases are transmitted to humans through mosquito bites. Thus, understanding mosquito biting behavior and obtaining biting rate estimates is important in the study of mosquito-borne disease dynamics. This study focuses specifically on *Aedes aegypti*, a vector for arboviruses such as dengue, Chikungunya, Zika, and yellow fever [2]. To examine how researchers have contributed towards understanding the population mean per mosquito biting rates of *Ae*. *aegypti*, we conducted a systematic review.

For decades, mathematical modeling studies have played an important role in understanding the dynamics of mosquito-borne diseases. The mosquito biting rate is a central parameter for modeling the transmission dynamics of mosquito-borne diseases and for estimating epidemiological parameters like the basic reproduction number and vectorial capacity. However, its empirical estimation is complex. Various methodologies have been used in the literature. An earlier systematic review [3] that studied available dengue transmission model structures critically assessed the underlying assumptions of these models based on epidemiological and entomological data. This earlier review provided a range of biting rate estimates used in mathematical modeling studies; however, the review aggregated results from different methodological approaches, making these estimates less comparable across other studies. In general, there is no standardized approach for estimating the biting rate parameter for *Ae*. *aegypti*. For example, many have estimated the per mosquito daily biting rate by taking the inverse of the mean gonotrophic cycle duration [4–7]. However, others have noted that, *Ae*. *aegypti* often bites more than once in a single gonotrophic cycle [8–13], suggesting that an adjustment is needed to capture the average number of bites per gonotrophic cycle. Some researchers have used mark-release-recapture (MRR) studies as their data source [14,15]; however, these studies are not specifically designed to estimate the biting rate.

Human landing catch (HLC) studies provide a rigorous estimate of the number of female mosquitoes landing on a human subject over a given time. Others have used this as an estimate of the number of mosquito bites per human [16], however, this estimate is strongly influenced by the number of humans used in the study. For example, if there is one human bait assigned to an HLC study in a house with 20 biting mosquitoes, there is the potential to estimate 20 mosquitoes/human-time. If there are 20 human baits, the estimate will be closer to 1 mosquito/human-time. Thus, HLC studies provide important information on relative biting rates but not the absolute measure. Given the relative measures obtained from these studies, they are most valuable for understanding variations in biting patterns and mosquito behaviors across factors such as temperature, season, and available diets. A few other modeling studies have obtained per mosquito biting rate estimates from non-human (e.g. Guinea pig) studies [15]; others simply assumed a value [17–19].

Mosquitoes’ biting behavior and rates are highly variable over time and space as well as across species and climates. To appropriately determine a biting rate value, this variability should be considered. Mosquito biting behavior varies across hours of the day [20–22], by location, for example, between indoors and outdoors [20–23], and across rural and urban settings [24]. Temperature is another factor that explains variation in mosquitoes’ daily biting rate. Lower temperatures slow down blood-meal digestion and lead to a longer gonotrophic cycle [25–27], which reduces mosquito’s daily biting rates. Based on temperature variations, the gonotrophic cycle duration can vary by up to four days [28]. Rainfall also has been shown to impact mosquitoes’ biting activities [20]. To better characterize the biting rate for *Ae aegypti* and its variability, we conducted a systematic review and compiled the available body of literature on the biting rate of *Ae*. *aegypti* under different contexts (e.g., season). Based on this review, we propose two viable approaches to estimate the population mean per mosquito biting rate: one considers multiple biting within a gonotrophic cycle and the other uses data from histological studies. To illustrate the use of these data, we provide a re-estimate of the biting rate based on data from one histological study [12] that collected the data required to estimate a population mean per mosquito biting rate.

## Methods

### Databases and search strategy

Two investigators (M.H.Z. and H.V.W.) searched ‘Pubmed’ and ‘Proquest’ for the following search terms: *Aedes aegypti*, *Ae*. *aegypti*, *A*. *aegypti*, biting rate, biting rates, biting frequency, biting habits, daily bites, blood feeding frequency, blood meals, frequency of feeding, feeding pattern, landing patterns, human bait, and diel biting (see S1 Text for all search terms with Boolean operators).

### Study selection

Studies included in this systematic review met the following criteria: (1) studied the biting rate or biting frequency of *Ae*. *aegypti*, (2) published in peer-reviewed journals, (3) published as a research article, not as a review article, (4) study conducted near or within a household or in a laboratory, (5) written in English or Spanish, and (6) published between January 1, 1970, and December 31, 2022. Initially, investigators reviewed the title and abstract only to assess whether articles met the inclusion criteria. Subsequently, investigators read the whole text of the manuscripts meeting our inclusion criteria.

**Fig 1:**
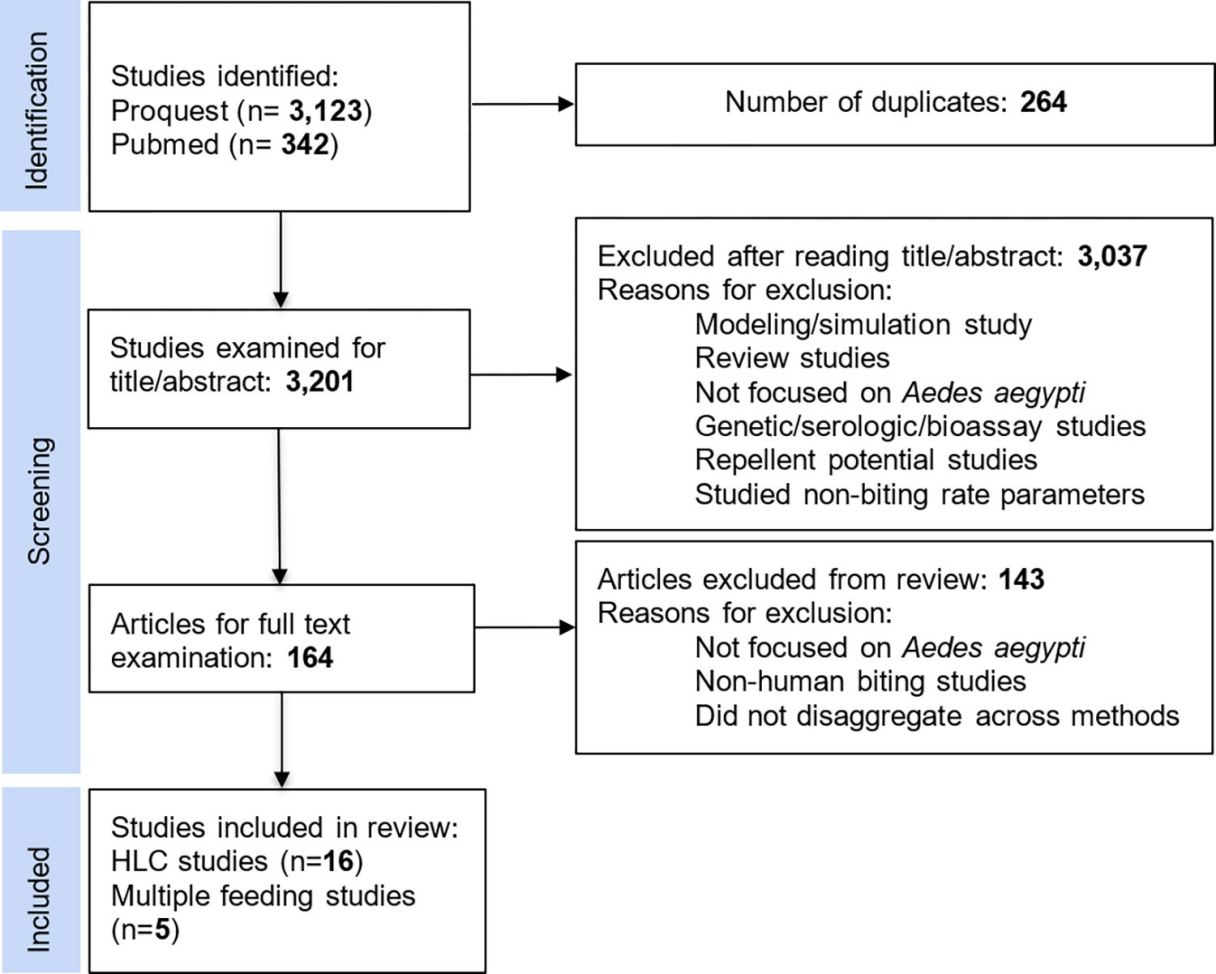
Flow chart depicting the process for exclusion and inclusion of articles.

A total of 21 studies were selected for detailed analysis (Fig 1). These 21 studies can be broken down into human landing catch studies (16) and multiple feeding studies (5) (Table 1).

**Table 1:** Categorization of studies included in this review.

	Human Landing catch (HLC) studies		Multiple feeding studies		
	Field studies	Lab studies	Histology	Mark-Release-Recapture (MRR)	DNA profiling
Number of studies	12	4	2	2	1

### Data extraction

For our analysis, we extracted biting data from five studies [21,23,29–31] that only provided data graphically using WebPlotDigitizer (https://apps.automeris.io/wpd/), a tool designed to extract data points from figures. One of the goals of this study is to investigate how climatic factors, such as rainfall and temperature impact biting of *Ae*. *aegypti*. However, only one [31] of the six field studies [21–23,29–31] that reported biting data also provided rainfall (graphically) and temperature data. For that specific study, we extracted the rainfall data using WebPlotDigitizer. For four [21,23,29,30] of the remaining five studies, we extracted temperature and rainfall data from the NASA Power Data Access Viewer (https://power.larc.nasa.gov/data-access-viewer/) over the timeframe of the study period. For temperature, we used the temperature at two meters (T2M) variable, the standard measurement for air temperature [32]. To extract these data, we obtained GPS coordinates for each study location from https://www.latlong.net/, provided in Table 2 for reproducibility. We could not extract temperature and rainfall data for the other study [23] because we did not find the study location in our GPS coordinate source (https://www.latlong.net/).

**Table 2:** GPS coordinates used to extract temperature and rainfall data.

Study	Study location	GPS coordinates (°N,°E)
Captain-Esoah et al. [21]	Bolgatanga, Ghana	(10.7148, -0.7661)
	Nadowli, Ghana	(10.3723, -2.6679)
	Damongo, Ghana	(9.0845, -1.8239)
Casas-Martínez et al. [22]	Tapachula, Mexico	(14.9109, -92.2648)
Salas-Luévano et al. [29]	Monterrey, Mexico	(25.6866, -100.3161)
Chompoosri et al. [30]	Central Thailand	(13.6306, 100.0547)

### Data analysis

One [21] of our reviewed studies reported hourly biting data averaged over three nearby locations. So, for [21], we estimate the mean temperature and rainfall values across these three locations (S1 Table). We also estimate seasonal biting for this study [21] averaging reported location-specific seasonal biting values (S2 Table). We use reported seasonal information and biting values to estimate the seasonal biting values for studies [30,31] (S4 Table). To maintain consistency in the mean biting values that we report, for one study [23], we converted their reported biting data from per night to per person-hour by dividing the per night biting rate by the number of hours and number of collectors (S3 Table). For another study [31], we converted the reported rainfall values from monthly to daily. S4 Table contains all biting data used in this manuscript.

## Results

We found two study designs that provide complementary information towards characterizing biting rate patterns. Each has their strengths and their limitations. First, the human landing studies: 1) characterize temporal variability at the hourly and seasonal scales, 2) characterize spatial variability (e.g., indoor vs. outdoor biting), 3) examine the extent to which climate factors (rainfall, temperature, and humidity) are determinants of biting activities, and 4) examine the extent to which host factors (host availability), vector factors, and environmental factors (diet availability) are determinants of biting activities. The other study design, multiple feeding studies, is implemented using two distinct methods, one a mark-release-recapture (MRR) approach and the other a histological approach. We present a review of biting rate estimates coming from these two study designs and the insights that they bring to our understanding of the biting rate and its variability. Studies reviewed in this study are summarized in Table 3. Finally, we propose two approaches to estimate the biting rate.

**Table 3:** Summary of studies reviewed in this study.

Study, Year	Study location	Study period	Rural/ Urban	Study type	Indoor/ Outdoor	Technique	Collection period & frequency
Thavara et al., 2001 [20]*.	Samui Island, Thailand	Mar, Jul 1996 Jan, Mar, May, Jun, Jul 1997 Jan, Jul 1998	Rural	HLC	Both	**Indoor**: 3 volunteers, each collected mosquitoes from their exposed legs ankle to knee in 2 dwellings in 7 villages. **Outdoor**: Similarly, 3 volunteers stationed themselves 15 meters away from the dwellings.	20 minutes, somewhere between 0800h-1200h for 2 consecutive days. Once a month
**Captain-Esoah et al., 2020 [21].**	Bolgatanga, Nadowli, and Damongo, Northern Ghana	Dry: Jan–Apr 2015, 2016 Rainy: Jul–Oct 2015, 2016	Not mentioned	HLC	Both	8 collectors (4 indoors and 4 outdoors) collected mosquitoes from their exposed lower legs in some rooms of 2 randomly selected houses.	0600–1800 hours, with 10-min breaks every hour over 3 consecutive days. Once a month, over an 8-month period
**Casas Martinez et al., 2013 [22].**	Tapachula, Mexico	March 2010 –January 2011	Urban	HLC	Both	6 tent traps per location (3 indoors and 3 outdoors) are used and one person inside the inner nets worked as a bait.	06:00 to 18:00 at intervals of 2 h, with 1h of collection and 1 h of resting. Twice a month, over a 9-month period
**Karch et al., 1995 [23].**	Bateképlateau, Zaire (DRC)	Feb 1990 –Nov 1991	Rural	HLC	Both	3 collectors in the same room and 3 others outside the house. The biting values were estimated from 1700 to 2100 hours because its activity fell off at 2100 hours.	1700–0600 hours Weekly collection
**Chadee & Martinez, 2000 [24].**	Port of Spain, Trinidad, West Indies	January–August 1999	Both	HLC	Both	For each of 2 houses, one researcher in the living room and another on the porch. Mosquitoes were caught with hand nets or aspirators from lower legs and ankles.	0400–2400 hours Once a week
**Salas-Luévano & Reyes-Villanueva, 1994 [29].**	Monterrey, Mexico	April–Oct 1991	Urban	HLC	Outdoor	2 volunteers captured landed mosquitoes with a portable electric vacuum cleaner. Mosquitoes were collected immediately after landing, during the inspection. Houses were made of wood with a sheet roof, which has ornamental plants and trees.	1600–1900 hours Once a week, over a 28-week period
**Chompoosri et al., 2012 [30].**	Central Thailand	Summer: Mar–May 2007 Rainy: June–Oct 2007 Winter: Nov 2007 –Feb 2008	Rural	HLC	Indoor	6 volunteers sat down on chairs with bared legs between knee and ankle in a row at a 5-meter distance from each other.	First 20-minute period of each hour over 24 hours period. Once a month
**Russell et al., 2005 [31].**	Moorea Island, French Polynesia	Sep–May 2003, 2004	-	HLC	Outdoor	Mosquitoes were collected by a person with an aspirator while sitting outside houses and other buildings in residential communities.	2 hours preceding sunset in each of 9 locations over 2 weeks. Once a month
**Dave D. Chadee, 1988 [33].**	La Seiva, Trinidad, West Indies	January–August 1980	Rural	HLC	Both	5 houses, one researcher in the living room and another on the porch. Mosquitoes were caught with hand nets or aspirators from lower legs and ankles.	0500–2000 hours Once a week
**Canyon et al., 2013 [37].**	Laboratory	-	-	HLC	-	One human host, biting was observed every 6 hours in low (34%RH) and high (84%RH) humidity environments.	10 minutes on each vial for 12 days
**Canyon et al., 1999a [39].**	Laboratory	-	-	HLC	-	One human host, biting was observed in 3 trials: every 6, 12, and 24 hours.	10 minutes on each vial for 12 days
**Canyon et al., 1999b [40].**	Laboratory	-	-	HLC	-	One human host, biting was observed every 6 hours in settings with 7 different diets.	10 minutes on each vial for 12 days
**Farjana & Tuno, 2013 [41].**	Laboratory	-	-	HLC	-	At 5–6 days after emergence, females were allowed to feed on human hands. Females who did not oviposit within 6 days after their first bloodmeal were allowed to take a 2nd bloodmeal, and if required, a 3^rd^ meal. All females, regardless of whether they had oviposited, were killed in a freezer to measure their wing length as an indicator of body size.	-
**Yasuno and Tonn, 1970 [34].**	Bangkok, Thailand	October 1967 –September 1968	Urban	HLC	Indoor	Two individuals, each collected mosquitoes from their legs while sitting in 4 rooms.	30-minutes. Once a week
**Nelson et al., 1978 [35].**	Jakarta, Indonesia	Jan-Mar 1975 (Wet season) Jul -Sep 1975 (Dry season)	Urban	HLC	Indoor	6 collectors collected mosquitoes landing on bare lower legs from 72 houses.	0600–1800 hours. Once a week
**Mutebi et al., 2022 [36].**	Miami, FL & Brownsville, TX	May–November 2019	Urban	HLC	Not mentioned	BG-Sentinel 2 traps were used to capture host-seeking female *Ae*. *aegypti* and monitored them every hour.	24 hours a day over 96 hours
**McClelland and Conway, 1971 [9].**	Dar es Salaam, Tanzania	04–27 June 1970	Suburb	Multiple feeding (MRR)	-	Female mosquitoes showing attempted biting behaviors were caught. After the 11th day, captured mosquitoes were merely preserved for checking.	5 hours period from dawn onwards. Once a day
**Trpis and Hausermann, 1986 [13].**	North Mombasa, Kenya	April–May 1972	Rural	Multiple feeding (MRR)	-	2 persons collected the mosquitoes landing on their exposed legs. Collected mosquitoes were marked and released between 1600 and 1700 hours in the same houses.	15 minutes Once a day
**Harrington et al., 2014 [8].**	Northwestern Thailand	Cool dry season: Feb 2000, 2001, 2002, and 2003 Warm rainy season: July 2000, 2001, and 2002	Rural	Multiple feeding (DNA profiling)	-	Human DNA blood meal profiling of the mosquitoes is used to quantify human-mosquito contacts. A total of 1,186 complete DNA profiles were used.	-
**Scott et al., 1993 [11].**	San Juan, Puerto Rico	March 1988	Urban	Multiple feeding (Histological)	Indoor	Mosquitoes were collected using backpack aspirators and then analyzed in the lab.	0800–1200 hours, Not mentioned
**Scott et al., 2000 [12].**	South Central Thailand and San Juan, Puerto Rico	June 1990 –June 1992 (Thailand) January 1991 –January 1993 (Puerto Rico)	Rural (Thailand), Urban (Puerto. Rico)	Multiple feeding (Histological)	Both	Aspirators in Puerto Rico and vacuum cleaners in Thailand are used inside, under, and around houses to collect mosquitoes, and then analyzed in the lab.	0800–1800 hours Once a week

*This study reported combine biting data of *Ae*. *aegypti* and *Ae*. *albopictus*.

### Hourly biting

**Fig 2:**
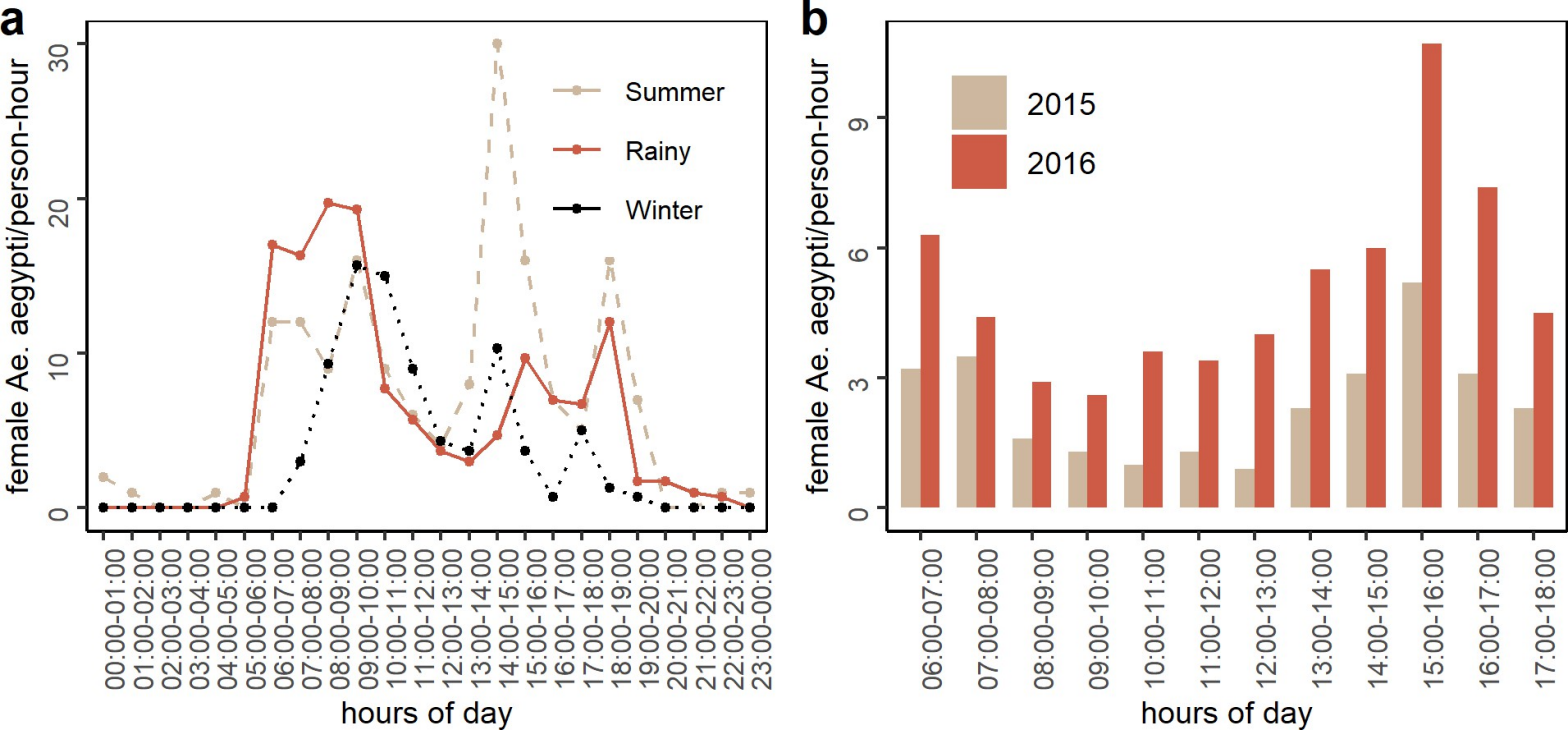
(a) Hourly biting activities across seasons reported by Chompoosri et al. [30], (b) Mean hourly biting reported by Captain-Esoah et al. [21] for 2015 and 2016.

We found two human landing catch (HLC) studies that reported *Aedes aegypti* indoor hourly biting (mosquitoes per human per unit time). Chompoosri et al. in Central Thailand [30] observed two biting activity peaks–one in the morning and the other in the afternoon during the rainy season and summer (Fig 2A). During the winter months, they observed a delayed morning peak with no clear afternoon peak. In Northern Ghana, Captain-Esoah et al. [21] also observed two peak biting periods during the day, both in the rainy and dry seasons. However, the mean hourly biting values reported in their manuscript were averaged across both rainy and dry seasons (Fig 2B). Two other publications monitored the hourly biting activity–one [20] reported a bimodal pattern estimating combined hourly biting activities of *Ae*. *aegypti* and *Ae*. *albopictus* (80.2% and 19.8%, respectively) on Samui Island, Thailand, while the other [22] did not observe any significant difference across the hours of the day while observing biting in rooms and yards in a city in Southern Mexico. Our review found four additional human landing catch (HLC) studies [33–36] that reported bimodal biting patterns without providing hourly data.

In 1999, Chadee and Martinez [24] observed a trimodal pattern comprising peaks one hour after sunrise, one hour before noon, and one hour before sunset. In this study, 10% of bites took place at night within urban areas, whereas no nighttime biting activity was reported within rural areas. On the other hand, an earlier study by Chadee (1988) [33] found that in a rural setting 5% of bites occurred at night.

### Seasonal biting

Based on available data, here, we reviewed three studies [21,30,31] to understand the biting activity and its variations across seasons. Some of these studies described three seasons (rainy, summer, and winter), while others reported two (rainy and dry or wet and dry). Two of these studies [21,31] provided monthly biting data along with seasonal information, while the other [30] provided hourly biting data across seasons. We used these data to estimate seasonal biting rates for these three studies (S2 Table) and identified that seasonal variations differed by study. One of these studies [21] showed that the mean biting values for the rainy season were 3.5 to 5-fold higher than during the dry season (S5 Table). Another study [31] found that biting rates increased during the wet season by 30% to 100%, depending on the geographic location along the coasts of Moorea Island, French Polynesia (S5 Table). The third study [30] observed that the biting activities during the summer were slightly higher than in the rainy season and the lowest during the winter. All estimated seasonal biting rates are tabulated in the S5 Table.

### Indoor–outdoor biting

**Fig 3:**
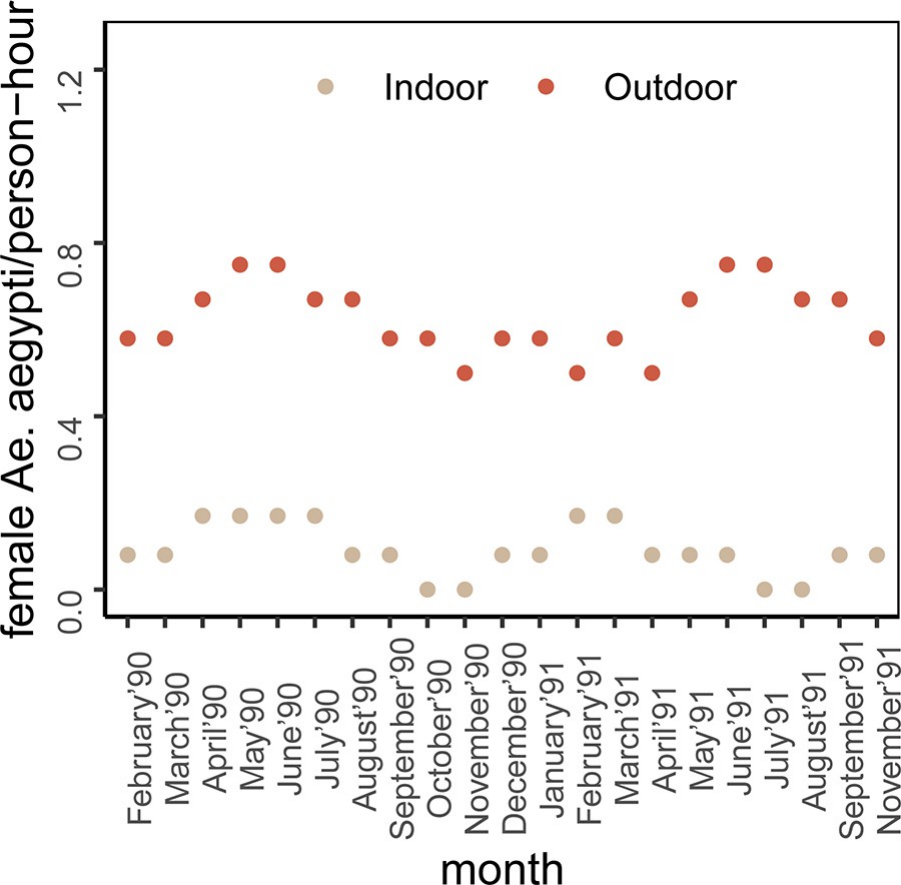
Indoor and outdoor biting activities between 1700–2100 hours in Northern Ghana (Karch et al. [23]). Here, the unit is converted from nightly to an hourly rate.

Our review identified six studies that reported indoor and outdoor *Ae*. *aegypti* biting activity. Four of them [21,23,24,33] reported higher biting activity outdoors compared to indoors (Fig 3 and S5 Table). This result was consistent for two [21,23] of these studies, regardless of seasonality. The other two studies [24,33] did not report monthly biting activities, so we could not look at these results stratified by season. This variation may be explained by higher mosquito densities in outdoor settings. In contrast to these four studies, Casas-Martínez et al. [22] observed higher indoor biting activities compared to outdoors, 4.10 female mosquitoes/human-day indoors compared to 1.88 female mosquitoes/human-day outdoors in Tapachula, Mexico. Another study [20] observed indoor-outdoor biting activities for *Ae*. *aegypti* and *Ae*. *albopictus* together and reported *Ae*. *aegypti* as primarily endophagic due to their rare presence outdoors.

### Impact of rainfall, temperature, and humidity

The rainfall and temperature data we used for this analysis was either reported in the reviewed manuscript or we collected from the NASA website (see data extraction subsection). Of the six studies [21–23,29–31] in this section, two [22,30] provided too few time points to analyze the relationship between weather patterns and biting activity. Our analysis of two of the remaining four studies provide mixed results. For example, we found using biting data from one study [23] that higher rainfall decreases indoor and outdoor biting activities (S1 Fig), however, using reported aggregated biting data across indoors and outdoors from another study [21], we found that higher rainfall increases biting activities (S2 Fig). With regards to temperature, we found in one study [23] that higher temperature decreases outdoor biting activities (S1B Fig), while in another study [21] we found that temperature had a non-linear relationship with biting activity (S2 Fig). Interestingly, the third study demonstrated a joint effect of temperature and rainfall. Salas-Luévano and Reyes-Villanueva [29] showed that biting frequency is higher either when the temperature is low (18° – 21°C) and the rainfall is moderate or when the temperature is high (25° – 28°C) and the rainfall is low (S3 Fig). Finally, Moorea Island in French Polynesia [31] observed the lowest biting activities on the west coast where rainfall was higher compared to the east coast. The temperature was similar on both coasts. In general, these studies suggest that there is no clear pattern between rainfall and temperature, and biting of *Ae*. *aegypti*.

A single study by Canyon et al. [37] investigated the impact of humidity in a lab setting and reported a moderate difference in biting frequencies in low (34% RH) versus high (84% RH) humidity conditions (0.077 and 0.083 per mosquito per hour at low and high humidity respectively; note the authors reported in units of per 6 hours resulting in the values 0.46 and 0.5). In their study, the temperature was constant and human blood (not human baits) was the only available diet. However, an earlier study [38] found a greater impact of humidity on biting activities due to the reduction in host-seeking activity at low humidity. Canyon et al. [37] suggested that this discrepancy was due to the difference in host availability, i.e., humidity may have a greater impact when hosts are less available. All biting activities discussed in this section were measured as female mosquitoes per person-hour.

### Impact of host availability

One study [39], conducted in a lab setting, examined how human host availability impacts the biting frequencies of *Ae*. *aegypti*. In their study, they made humans available to mosquitoes every 6, 12, and 24 hours and estimated the per female mosquito biting frequencies as 0.47 per 6 hours, 0.54 per 12 hours, and 0.7 per 24 hours, respectively (or equivalently, 0.08, 0.045, and 0.03 per hour). Based on these results, the authors concluded that *Ae*. *aegypti* are opportunistic feeders, i.e., increasing host availability results in increased biting.

### Impact of diet availability

Another lab study [40] investigated the impact of diet availability for four diets (blood + 10% sugar, blood + 3% sugar, blood + water, and blood only) on human-biting. The study observed that the availability of sucrose reduced host biting frequencies by up to 50%, based on available sucrose (median probes/bites per mosquito per hour: 0.037 for the ‘blood + 10% sugar’ diet, 0.047 for the ‘blood + 3% sugar’ diet, and 0.083 for the blood-only diet which were originally reported as median probes/bites per mosquito per 6 hours: 0.22 for the ‘blood + 10% sugar’ diet, 0.28 for the ‘blood + 3% sugar’ diet, and 0.50 for the blood-only diet). To a lesser extent, the availability of water also decreased the biting frequencies (median probes per mosquito per hour: 0.075 for the ‘blood + water’ diet compared to 0.083 for the blood-only diet which were originally reported as per mosquito per 6 hours: 0.45 for the ‘blood + water’ diet compared to 0.50 for the blood-only diet). These investigations were carried out at high relative humidity conditions (84% RH).

### Impact of mosquito body size

Two of our reviewed studies examined the relationship between mosquitoes’ body size and their biting frequency on humans. Scott et al. [12] found an inverse correlation in Thailand, where temperatures were higher, whereas almost no relation in Puerto Rico where temperatures were lower overall. The other study by Farjana and Tuno [41] found no correlation in their lab study.

### Multiple feeding

We identified five studies that reported multiple feeding of *Ae*. *aegypti* in a single gonotrophic cycle. Two of them are mark-release-recapture (MRR) studies. McClelland and Conway [9] released 418 singly marked female mosquitoes, of which 48 were recaptured once and 5 were twice, among which 7 double-fed within 24 hours. Trpis and Hausermann [13] released 563 uniquely marked female *Ae*. *aegypti* of which 272 (48.3%) were recaptured. Of these 272 recaptured, 47.8% were recaptured once, one was recaptured 10 times, and the remaining were recaptured anywhere between two and seven times. Based on these values and the measured developmental stages of their ovaries, existing blood in their stomachs, and estimated survival rate, they concluded that most *Ae*. *aegypti* bite twice, and some three times within a gonotrophic cycle.

Histological techniques and DNA profiling of human blood have also been used to study the feeding frequency of *Ae*. *aegypti* within a gonotrophic cycle. Two histological studies by Scott and his colleagues [11,12] reported multiple feeding of *Ae*. *aegypti*. They diagnosed field-collected *Ae*. *aegypti* mosquitoes and reported that many mosquitoes fed twice on the same day or by the following day. Harrington et al. [8], using DNA profiling of human blood, found that 10–13% of engorged mosquitoes fed on more than one person in a 24-hour period.

### Importance of accounting for multiple feeding

Multiple feeding studies are crucial to estimate per mosquito biting rate because they challenge the classic approach of estimating biting rate as the inverse of the gonotrophic cycle duration (GCD). One multiple feeding study [13] identified that the majority of *Ae*. *aegypti* fed more than once within a gonotrophic cycle. Thus, an assumption that at least 50% feed at least twice results in a mean of 1.5 bites per GCD. Several recent studies [42,43] studying *Ae*. *aegypti* across the globe reported the duration of the gonotrophic cycle as between 3 to 4 days. Based on these values, the traditional classic approach that ignores multiple feedings estimates the per mosquito biting rate to be between 0.25 and 0.33 (¼ and 1/3) bites/day while accounting for multiple feedings estimates of 0.38 to 0.5 (1.5/4 to 1.5/3) bites/day, which is 50% higher than the traditional estimate.

The mark-release-recapture (MRR) and histological studies are commonly used to track and estimate multiple biting of *Ae*. *aegypti;* however, only MRR studies are able to track multiple biting within any given period, whereas the histological studies can only track 48 hours prior to capture. With estimates of the gonotrophic cycle duration and multiple feeding per gonotrophic cycle, we can estimate the mean per mosquito biting rate (MBR) for any mosquito population using the following equation:

$$MBR=\frac{mean\;\#\;of\;bites\;per\;mosquito\;within\;a\;gonotrophic\;cycle}{Gonotrophic\;Cycle\;Duration\;(GCD)}$$

Research shows that lower temperatures slow blood-meal digestion and lead to a longer gonotrophic cycle [25–27]. This variability in the GCD and the influence of multiple feeding on the biting rate estimates illustrate the importance of using regionally specific estimates.

### Biting rate estimation

Of the 21 manuscripts that we reviewed, only Scott et al. [12] provides the necessary data to estimate per mosquito biting rates. They provide both the number of feedings within the past 2 days or more, and the interval between their feeding time and the day of capture for each captured mosquito. Specifically, Scott et al. [12] collected mosquitoes once a week over a 2-year period in both Thailand and Puerto Rico. Tables #4 and #5 in their manuscript [12] describe the data they collected relevant to estimating the per mosquito biting rate. In Table #4 found in [12], we observe that in Thailand, 595 out of 1,300 mosquitoes fed on the day of capture of which 255, 310, and 30 fed one, two, or three times respectively, whereas in Puerto Rico, 548 out of 1,156 mosquitoes fed on the day of capture of which 293, 244, and 11 fed one, two, or three times respectively. The table #4 also provides data on the number of mosquitoes that had their last blood meal on days other than the day of capture. In Table #5 found in [12], we observe that in Thailand, 65% of mosquitoes that fed twice took both blood meals on the same day, and 78% of mosquitoes that fed three times took their last two blood meals on the same day. In Puerto Rico, 43% of mosquitoes that fed twice took both blood meals on the same day, and 82% of mosquitoes that fed three times took their last two blood meals on the same day. Using these data, they estimated the average number of feeding events at 0.76 and 0.6 per mosquito per day in Thailand and Puerto Rico, respectively.

We use the same dataset to re-estimate the average per mosquito biting rate based on a different interpretation of the data. For our calculations, we assume that biting and feeding rates are equivalent. To estimate a per mosquito biting rate, we relied on the number of bites the mosquitoes took on the day of capture (Tables #4 and #5 in [12]). In the Thailand study, 255 mosquitoes bit only once, and that bite occurred on the day of capture. Also, 310 mosquitoes bit twice where the last bite was on the day of capture (Table #4 in [12]), of which 65% had both their meals on the day of capture (Table #5 in [12]) and the remaining 35% had only the last bite on the day of capture. Finally, they reported 30 mosquitoes that bit three times where the last bite was on the day of capture. Since Scott et al. [12] did not report any mosquitoes having all three meals on the same day, 78% of mosquitoes that had three bites (30 mosquitoes) had their 2nd and 3rd bites on the day of capture and the remaining 22% had only their 3rd bite on the day of capture. In Table #5 in [12], 61% of mosquitoes that took three bites in total are listed as having taken their first two bites on the same day. We assumed that those mosquitoes could not have taken those bites on the day of capture since no mosquitoes were identified as having taken all three bites on the day of capture.

Based on these data, we first estimated the total number of bites taken on the day of capture (Table 4 in this manuscript). We then multiplied this number by 0.95 and 0.97 for Thailand and Puerto Rico, respectively, because 95% of engorged *Ae*. *aegypti* in Thailand fed on humans (88% fed on human blood only and 7% fed on mixed blood) and 97% of engorged *Ae*. *aegypti* in Puerto Rico fed on humans (95% fed human blood only and 2% fed on mixed blood) [26]. Next, we divided by the total number of engorged mosquitoes to calculate the average per engorged mosquito daily biting rate (Table 4 in this manuscript). Finally, we adjusted our estimates because only engorged mosquitoes were captured and analyzed. Scott et al. [26] estimated that 59 and 58% of captured female mosquitoes were engorged in Thailand and Puerto Rico, respectively. The same study also reported data on gravid mosquitoes and gut contents. In this estimation, we assumed that a portion of the non-engorged mosquitoes, those that were non-gravid and had non-empty gut contents, also bit at the same rate as the engorged mosquitoes. We also assumed that the remaining (gravid or empty) did not bite during the observation period.

**Table 4:** Per mosquito biting rate estimation for Ae. aegypti using data from Scott et al [12]. The data represent engorged mosquitoes captured on a single day (595 of 1,300 from Thailand and 548 of 1,156 from Puerto Rico). These mosquitoes were grouped into those in which the histology analysis indicated the mosquito bit one, two or three times. The total number of bites that occurred on the day of capture were tallied (Column 2).

	Number of mosquito bites on day of capture	Human-bites per day*	Notes
**Thailand**		(95% of all bites)	
Mosquitoes that bit once (255)	255	242.25	All bites on the day of capture.
Mosquitoes that bit twice (310)	511.5	485.93	65% (= 201.5) of double biters took both bites on the day of capture and so we count 2 bites for these mosquitoes; the remaining 35% (=108.5) took only the last bite on the day of capture and so we count 1 bite for these mosquitoes.
Mosquitoes that bit three times (30)	53.4	50.73	78% (=23.4) of triple biters took their last two bites on the same day, (here, the day of capture), and so we count 2 bites for these mosquitoes; the remaining 22% (= 6.6) took only the last bite (3rd) on the day of capture and so we count 1 bite for these mosquitoes.
Total	819.9	778.91	
Average biting per mosquito per day = (778.91 /1,300) x 69% = 0.41			
**Puerto Rico**		(97% of all bites)	
Mosquitoes that bit once (293)	293	284.21	All bites on the day of capture.
Mosquitoes that bit twice (244)	348.92	338.45	43% (= 104.92) of double biters took their last two bites on the same day, (here, the day of capture), and so we count 2 bites for these mosquitoes; 57% (=139.08) took only the last bite on the day of capture and so we count 1 bite for these mosquitoes.
Mosquitoes that bit three times (11)	20.02	19.42	82% (= 9.02) of these triple biters took two bites on the day of capture and so we count 2 bites for these mosquitoes; the remaining 18% (= 1.98) took only the last bite (3rd) on the day of capture and so we count 1 bite for these mosquitoes.
Total	661.94	642.08	
Average biting per mosquito per day = (642.08 /1,156) x 63% = 0.35			

* Human-bites per day (3rd column) is 95% and 97% of the number of mosquito bites on day of capture (2nd column) for Thailand and Puerto Rico, respectively. i.e., for Thailand, 3rd column = 95% of the 2nd column and for Puerto Rico, 3rd column = 97% of the 2nd column.

Based on data presented in [26], we estimated that among the non-engorged mosquitoes, 41% and 42% (100–59% and 100–58%) in Thailand and Puerto Rico, respectively, 44% and 31% (100–56% and 100–69%) respectively were reported as non-gravid. Thus, the percentages of non-engorged mosquitoes that were both not gravid and not empty in Thailand and Puerto Rico, respectively were 10% (0.41*0.44*0.56) and 5% (0.42*0.31*0.39). The biting mosquitoes are consist of engorged mosquitoes and the non-engorged mosquitoes that are neither gravid nor empty. Thus, the overall percentages of mosquitoes that were biting were therefore 69% (59% + 10%) and 63% (58% + 5%) respectively. We used these numbers to adjust the biting rate estimates, originally measured for engorged mosquitoes, to the total mosquito population (Table 4 in this manuscript).

Our approach estimates biting rates of 0.41 and 0.35 bites per mosquito per day in Thailand and Puerto Rico, respectively. In comparison, Scott et al. [12] estimated the biting rates as 0.76 and 0.63 respectively. The difference comes about because for mosquitoes that bite twice, they counted all mosquitoes that had both bites on the same day instead of only those mosquitoes that had both bites on the same day and the last bite on the day of capture i.e., for Thailand, 65% of 42% (541 of all mosquitoes) instead of 65% of 24% (310 of all mosquitoes) while for triple-biters, they counted mosquitoes that bite three times and imbibed any two consecutive meals (1st and 2nd or 2nd and 3rd) on the same day instead of counting only those mosquitoes that had the last bite on the day of capture and had last two meals on the same day i.e., for Thailand, 61% and 78% of 5% (60 of 1,300) mosquitoes instead of only 78% of 2.5% (30 of 1,300) mosquitoes. Moreover, they did not consider those non-engorged mosquitoes that were neither gravid nor empty. To illustrate the difference between these two approaches, we represent our estimation in a similar fashion as of Scott et al. [12]. For Thailand, our estimate is:

0.41=(0.95×(0.46+[0.24×0.65]+[0.025×0.78]))×69%

For Puerto Rico, our estimate is:

0.35=(0.97×(0.48+[0.21×0.43]+[0.01×0.82]))×63%

## Discussion

The mosquito biting process is highly variable and dynamic. Rates over vary numerous factors, including mosquito density and hour of collection, and is therefore difficult to characterize. We found several study designs that contribute to the understanding of biting patterns and provide data to estimate rates under different contexts. First, human landing catch studies, both field and lab, capture key determinants of variability in *Ae*. *aegypti*’s biting rates and its variability across spatial, temporal, and environmental factors. These studies are helpful to understand relative biting activities; however, these studies estimate the number of mosquitoes landing per human per time, while the biting rate is the number of bites per mosquito per unit time. Second, the inverse of the gonotrophic cycle is often used as a biting rate estimate, however, this approach is valid if mosquitoes bite only once in a gonotrophic cycle. Finally, histological studies are promising in their ability to generate data useful for estimating per mosquito biting rates. Here we discuss the strengths and limitations of each of these approaches.

The strength of human landing catch (HLC) studies is their ability to provide information on relative biting behavior across a variety of factors. This review captures interesting characteristics across publications, including the potential for trimodal biting patterns in both rural and urban areas [24] and significant non-diurnal (nocturnal) biting [24,33], especially in urban areas [24]. It also captures the observation that, on average, outdoor biting is higher than indoors (Fig 3 and S5 Table). We found that seasonality, rainfall, and temperature affected biting activities, while humidity did not. One of our reviewed studies suggested a non-linear relationship between biting frequency and temperature, which was also identified in earlier studies [44,45]; however, the combined impact of temperature and rainfall on mosquito biting has not been studied. Our summary of three studies [21,30,31] suggests that outdoor biting activity is higher in the rainy or wet season compared to other seasons which is not always true for indoor biting activity (S5 Table). Comparisons across studies were often difficult due to the differences in study designs. For example, the collection periods in human landing catch studies varied from 15 to 60 minutes. Developing standardized study designs would make it easier to compare estimates across studies.

Obtaining biting rate estimates using the inverse of the gonotrophic cycle is a relatively common approach because these data are readily available. Since *Ae*. *aegypti* often feeds more than once per gonotrophic cycle, this often-used approach will underestimate the biting rate. Thus, additional information is needed from multiple feeding studies to obtain estimates of the average number of bites occurring within a gonotrophic cycle. A prior review study [46] has acknowledged the importance of accounting for multiple feeding in determining the mosquito biting rate that we summarized in the equation above. In their review, they challenge the common assumption of one feeding per cycle, providing evidence that many mosquito species, including *Ae*. *aegypti*, feed more than once per gonotrophic cycle.

Histological studies are capable of identifying the number of blood feeds that an engorged mosquito has undertaken and the timing of its last feeding. These studies are also capable of detecting intervals between subsequent feedings. Thus, histological studies provide a lot of information towards estimating biting rates. One limitation of this approach is that histological detection is only possible a few days prior to capture, thus limiting the time interval from which to count bites [12]. It also cannot detect interrupted feeds (i.e., when a mosquito feeds on one person, is swatted away, and then feeds immediately on someone else). So, the estimation of daily biting rates using data from histological studies will tend to underestimate the actual value. Despite these limitations, histological studies provide one of the best empirical datasets to estimate mosquito biting rates.

We suggest that all three of these study designs (human landing catch, marked-release-recapture, and histological) play an important role in estimating per mosquito biting rates and capturing at least some of its variability. The primary goal of this review was to systematically review the literature for data that can be used to estimate the per mosquito biting rate of *Ae*. *aegypti*. We found a few relevant studies that could be used to estimate per mosquito biting rates, limiting our ability to provide summary estimates of the biting rate. The huge variability in mosquito biting behavior is also a major limitation to obtaining robust biting rate estimates. However, examining estimates across different study designs and highlighting studies that characterize variability strengthens our understanding and confidence in results. Factors like rainfall, temperature, and diet availability affect the biting behavior of *Ae*. *aegypti* and should be considered in estimates when possible. Further exploration is needed to characterize how climate affects other related variables, such as the duration of gonotrophic cycles.

In our review we found the number of studies capturing the biting rate variability of *Ae*. *aegypti* across important environmental factors to be limited. Specifically, we found only six studies [20–24,33] that compared indoor vs. outdoor biting, one study [24] that compared urban and rural, three studies [20,21,30] that provided hourly biting patterns within a day, three studies [21,30,31] examining seasonal biting patterns within a year, and only one study [37] that examined the impact of humidity. We also found two field studies [20, 31] that reported rainfall data and only one study [31] that reported temperature data. More data are needed to fully characterize how environmental factors impact the variability of per mosquito biting rates.

Biting rates are highly variable over space and time. One component of this variability comes into play when multiple factors determine the biting rate. In our proposed method, gonotrophic cycle duration and the number of feeds a mosquito has within one gonotrophic cycle can contribute to this variability if the sources of these data come from different contexts. Other sources of any residual variability are highlighted in the human landing catch studies that estimate variation by season, location (e.g., outdoor vs. indoor), and time of day among others. A range or distribution of estimates provide a means for transmission modeling studies to incorporate this variability in analyses to increase the robustness of their conclusions. In general, transmission modeling studies should be more informed by the biology of mosquito behavior [46]. By understanding the biology of blood-feeding and context-specific factors, we can arrive at more informed per mosquito biting rate estimates for site-specific transmission model analysis.

## Supporting information

S1 Text Search terms with Boolean operators. (DOCX)

S1 Table Temperature and rainfall estimation for Captain-Esoah et al. study [21]. (XLSX)

S2 Table Seasonal mean biting estimation. (XLSX)

S3 Table Mean biting estimation for Karch et al. study [23]. (XLSX)

S4 Table Biting and other relevant data used in this study. (XLSX)

S5 Table Seasonal mean biting values. (XLSX)

S1 Fig Impact of rainfall and temperature on indoor-outdoor biting activity. (TIF)

S2 Fig Impact of rainfall and temperature on biting activity across years. (TIF)

S3 Fig Impact of rainfall and temperature on outdoor biting activity. (TIF)
