# Supplementary material for: The biting rate of Aedes aegypti and its variability: A systematic review (1970–2022)
Source: PLoS Negl Trop Dis. 2023 Aug 8;17(8):e0010831. doi: 10.1371/journal.pntd.0010831 (PMC10456196; doi:10.1371/journal.pntd.0010831)
Supplement: S1 Text — (DOCX) [file pntd.0010831.s001.docx]

("Aedes aegypti" OR "Ae.aegypti" OR "A. aegypti") AND ("biting rate" OR "biting rates" OR " biting frequency" OR "biting habits" OR "daily bites" OR "blood feeding frequency" OR “blood meals” OR "frequency of feeding" OR "feeding pattern" OR "landing patterns" OR "human bait" OR “Multiple blood feeding” OR “diel biting”)
