# Supplementary figures and images for: The biting rate of Aedes aegypti and its variability: A systematic review (1970–2022)
Source: PLoS Negl Trop Dis. 2023 Aug 8;17(8):e0010831. doi: 10.1371/journal.pntd.0010831 (PMC10456196; doi:10.1371/journal.pntd.0010831)

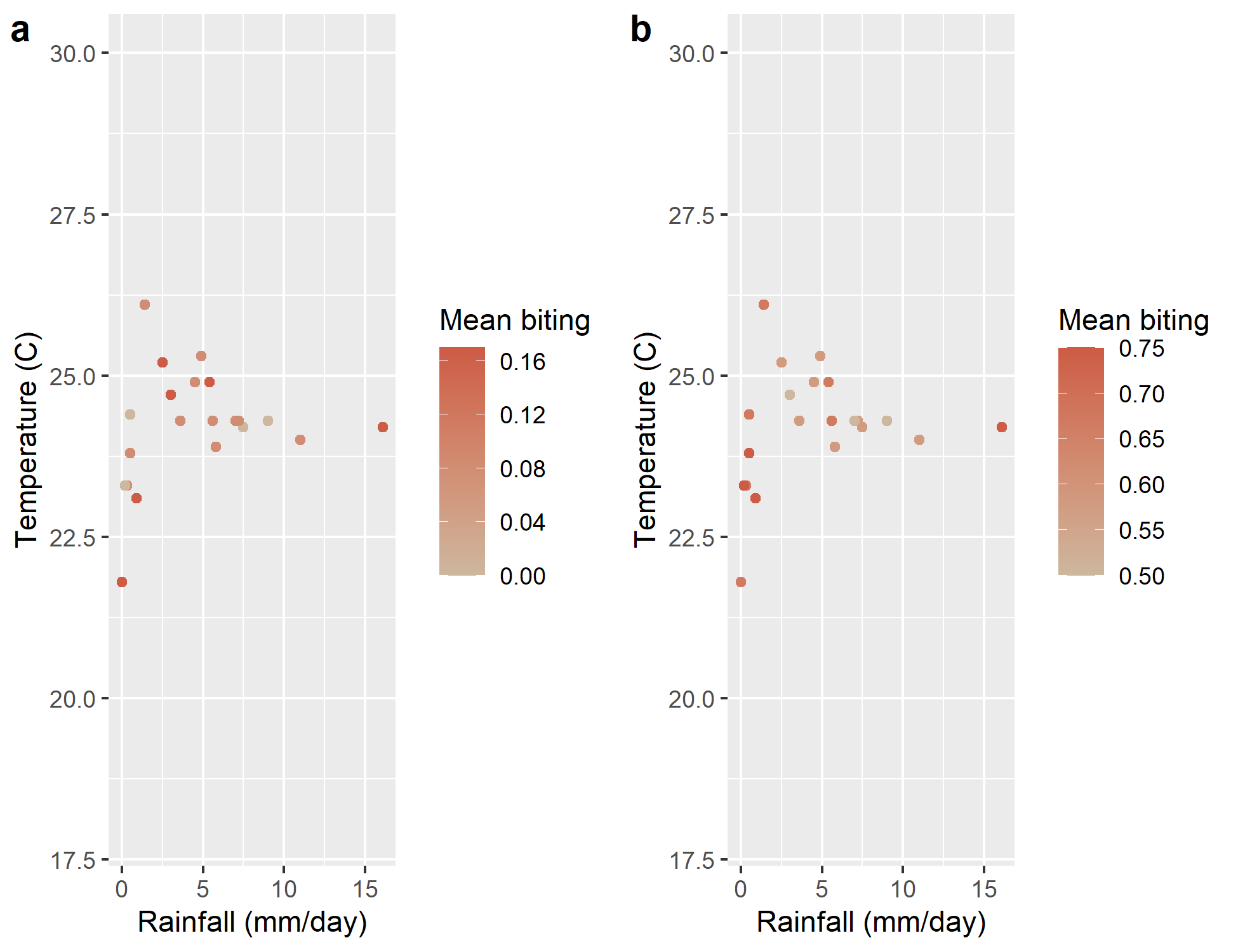

Supplement: S1 Fig — (TIF) [file pntd.0010831.s007.tif]

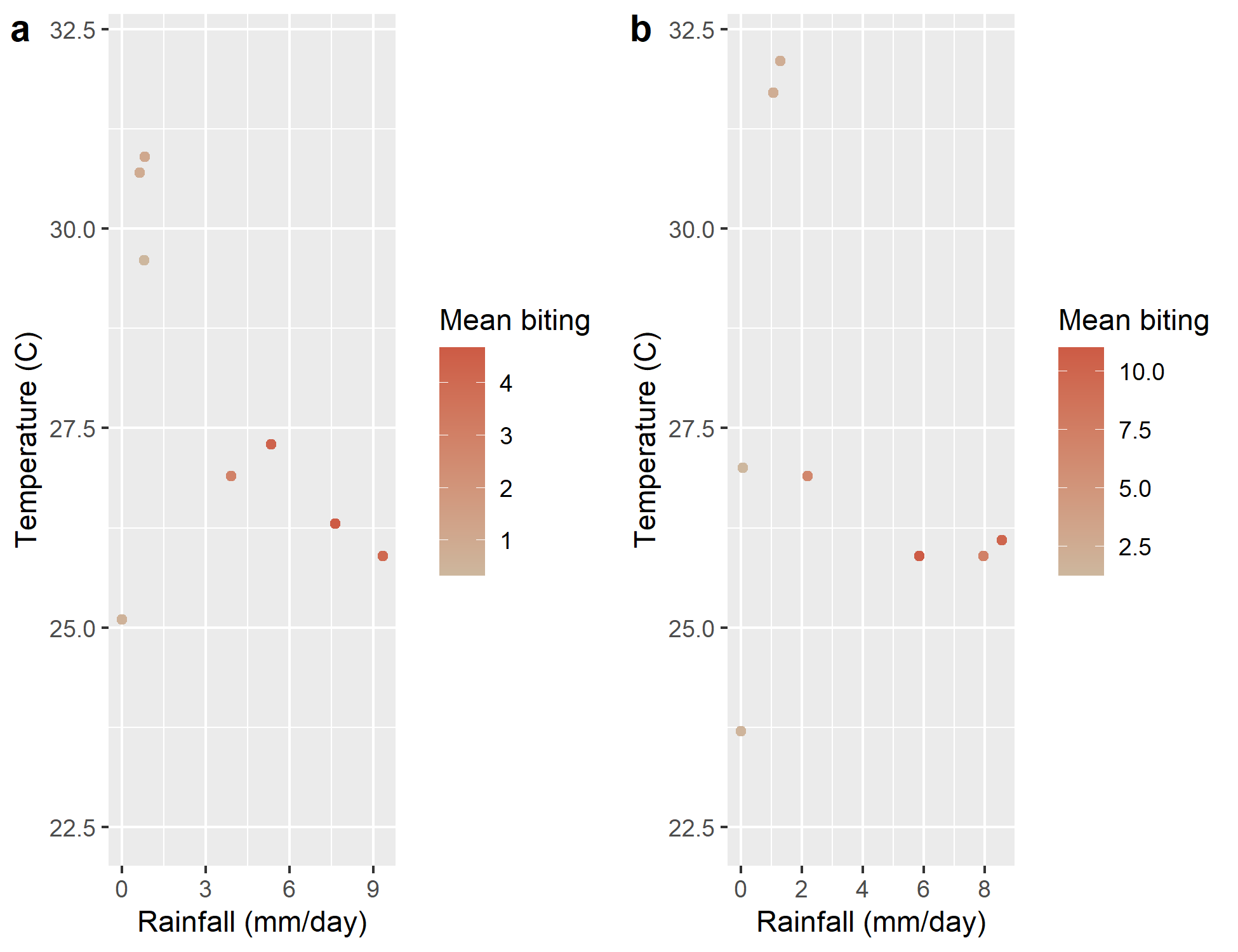

Supplement: S2 Fig — (TIF) [file pntd.0010831.s008.tif]

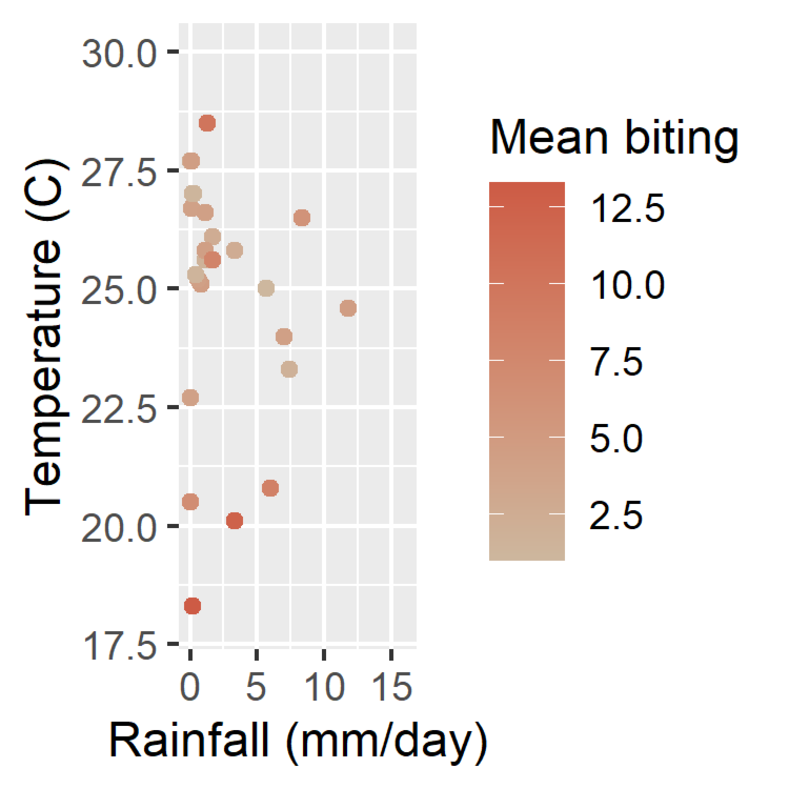

Supplement: S3 Fig — (TIF) [file pntd.0010831.s009.tif]
